# Production, acceptability, nutritional and pasting properties of orange-flesh sweet potato, cowpea and banana flour mix

**DOI:** 10.1038/s41598-024-55312-1

**Published:** 2024-02-26

**Authors:** Abiola Folakemi Olaniran, Clinton Emeka Okonkwo, Omorefosa Osarenkhoe Osemwegie, Yetunde Mary Iranloye, Adejoke Deborah Adewumi, Abiola Ezekiel Taiwo, Oluwakemi Christianah Erinle, Iyanuoluwa Esther Ajayi, Oluwafemi Adeleke Ojo

**Affiliations:** 1 Department of Food Science and Microbiology, College of Pure and Applied Sciences, Landmark University, P.M.B. 1001, Omu-Aran, Kwara State Nigeria; 2 Department of Agricultural and Biosystems Engineering, College of Engineering, Landmark University, P.M.B. 1001, Omu-Aran, Kwara State Nigeria; 3 Department of Food Science, United Arab Emirates University, Al Ain, United Arab Emirates; 4 Faculty of Engineering, Mangosuthu University of Technology, 511 Griffiths Mxenge Highway, Durban, South Africa; 5 Department of Biochemistry, Bowen University, Iwo, 232101 Osun State Nigeria

**Keywords:** Orange fleshed sweet potato, Fortificant, Fermented paste, Complementary food, Vitamin A, Biochemistry, Biochemistry

## Abstract

Promoting the intake of foods rich in vitamin A is key to combating the increase in vitamin A deficiency**.** This research focused on the utilization of orange-fleshed sweet potatoes (a tuber-based food), cowpea (a pulse), and ripe bananas (a fruit) for the production of flour mix as a means to reduce Vitamin A deficiency in children. Different ratios of sweet potato-cowpea-banana (PCB) mix, resulting in 8 different blended samples, were optimized. The flour mix was evaluated for its overall acceptability, vitamin A content, beta-carotene, and other nutritional and functional properties. The panelists rated the sweet potato-cowpea banana blends labeled PCB_8_ (60% OFSP, 30% cowpea, 5% ripe banana flour, and 5% sugar) as most preferred and acceptable with average scores of 8.96 points for color, 8.75 points for flavor, 8.88 points for appearance, 8.33 points for taste, 8.07 points for texture, and 8.39 points for overall acceptability on a 9-point hedonic scale. The vitamin A and beta-carotene contents ranged 7.62 to 8.35 mg/100 g and 0.15–0.17 mg/100 g for all blends. A significant difference in the functional properties of the flour mix were observed with an increase in the ratio of sweet potato flour addition. Findings from this study show that the flour mix PCB_4_ (65% sweet potato, 30% cowpea, and 5% ripe banana flour) was acceptable (8.15) and is recommended based on its vitamin A content (8.35 mg/100 g), nutritional properties, and functional properties. The study showed that locally available food commodities have good nutritional value that will help reduce vitamin A deficiency in children.

## Introduction

In sub-Saharan Africa, parents are becoming more worried about nutritional deficiency in their young children, since it continues to persist due to dietary pattern^1^. Vitamin A deficiency (VAD) is the leading cause of preventable blindness, affecting many children and women of reproductive age^2^. VAD affects 40–60% of African children and 10–20% of pregnant women in low-income countries^3^. Vitamin A is essential for immune system functions and the survival, growth, and development of children^4^. Hence, promoting the intake of provitamin A-rich foods is open to a diversity of foods and can be used to enhance the former strategies^5^. Roots and tubers are generally one of the cheapest and most popular sources of dietary energy in the form of carbohydrates^6,7^. Orange-fleshed sweet potato (OFSP) is naturally rich in vitamin A, especially β-carotene, and a good source of starch, sugars, and minerals. OFSP is an emerging tuber-based food for the improvement of vitamin A status in the world, particularly in developing countries. It was acknowledged as an excellent source of natural health-promoting compounds, diverse vitamins (A precursors), and minerals that can meet nutrient intake and reduce VAD^8^. Studies have shown that consumption of OFSP has the potential to curtail VAD which is prevalent in children leading to early blindness and death of a minimum of two hundred and fifty thousand children in Africa^9^. They serve as a major inexpensive source for improving immunity, preventing blindness in children and women of reproductive age, combating widespread VAD, and reducing degenerative diseases like cancer, cardiovascular disease, cataracts, muscular degeneration, etc^10–12^. Existing studies on the formulation of products have been found to utilize sweet potato in combination with wheat, rice, soybean, cowpea, peanut, cassava, and so on^2,7–9,13^.

The utilization of pulses in the diet has a healthy approach to lessening the risks of several chronic disorders and helping in meeting dietary recommendations^14^. Commonly used legumes throughout the world are beans, chickpeas, lentils, peas, cowpeas, broad beans, and soybeans^15^. Cowpea (*Vigna ungucuilata*) is a primary leguminous food, with about 90% being produced in the West Africa region of the world^16^. The largest producer of cowpea is Nigeria, which accounts for 61% of production in Africa and 58% worldwide^17^. Cowpea seeds contain a high protein content (18 to 25%), 1.4–2.7% of fats, and about 6% crude fiber. It also contains about 7.5 mg per 100 g of iron and contains non-essential and essential amino acids, e.g., valine, leucine, phenylalanine, and lysine. The calcium content of cowpeas is higher than that of meat (7%). Besides, it has also been reported as an important source of protein for a large part of the world’s population, especially in countries with the poorest populations and high rates of protein-energy malnutrition^17^. Cowpea contains water-soluble vitamins (thiamine, riboflavin, and niacin) comparable to the levels found in fish and lean meat^18^, thus making it extremely valuable to help in alleviation of protein deficiency.

The banana (*Musa* *acuminata),* when ripe, is consumed raw as a dessert fruit, which serves as a good source of carbohydrates, minerals such as potassium, and vitamins^19^. Biernacka et al.^20^ reported that they contain many important vitamins (A, C, E, K, and B groups), are rich in fiber, and contain many minerals (magnesium, phosphorus, calcium, and potassium). It was also reported to ease constipation due to its fiber content and prevent anemia by stimulating the production of hemoglobin due to its iron content^21^; about 25% of the total food energy for about 60 million people in Africa comes from bananas and plantains^22^. Nigeria is one of the largest banana-producing countries in Africa and accounts for about 2.73 million metric tons of bananas per year^23^. However, during glut season, ripe banana wastes in tonnes due to poor handling and inadequate storage facilities^24^.

Hence, the production of banana flour from ripe ones would reduce the post-harvest loss of bananas and contribute to the attainment of SDG goal 12, aimed at sustainable responsible consumption and production, thus converting them into useful products. Thus, to combat VAD prevalence and meet the description of being nutritious, acceptable, assessable, and cheap, this study explored the use of orange-flesh sweet potatoes as a source of vitamin A, cowpea, and ripe bananas as sources of protein and iron, respectively, in the production of the flour mix.

## Result and discussion

### Nutritional composition of the formulated sweet potato, cowpea-banana blends

The nutritional composition of the formulated PCB blends is presented in Tables 1, 2, 3. There was an important significant difference (*P* < 0.05) in the nutritional composition of the formulated blends. Moisture content value ranged from 2.44 (PCB_8_) to 3.80% w.b (PCB_7_). The ash content of the PCB blends varied between 4.04 and 5.14%. It was found to be highest in PCB_4_, while PCB_8_ presented the lowest ash value, followed by PCB_8_. PCB_6_ showed the lowest fiber content (1.74%), which agrees with the reduction in the proportion of the orange-fleshed sweet potato (OFSP) and banana in the blend, conversely, PCB_1_ showed the highest fiber content followed by PCB_8_, PCB_3_, and PCB_4_. An increase in fiber as the addition of OFSP flour increases is similar to the observations of Kidane et al.^13^ and Afework et al.^8^ during the production of bread and pasta, respectively. Consumption of high-fiber food products has been linked to a reduction in hemorrhoids, diabetes, high blood pressure, and obesity^21^. Small variations were observed in the fat content of the blends; it ranged from 0.62 to 0.88 percent. The PCB_5_ blend had the highest fat content, while the PCB_4_ blend had the lowest fat content. Fat content has been reported to play a crucial role in the shelf-life stability of flour products^25^. There was a slight variation in the protein content of the formulated blends; it varied between 8.46 and 10.52%. PCB_5_ presented the highest protein value, which is mainly derived from the increase in cowpea content. The total carbohydrate content of the blends ranged from 77.89% to 81.33%. PCB_8_ blend had the highest carbohydrate content, closely followed by PCB_7_ (80.33%), PCB_6_ (79.90%), and PCB_3_ (79.58%), though there was no significant difference between them. The higher value of carbohydrates was due to an increase in the proportion of the OFSP inclusion. The energy content of the blends ranged from 1331.36 to 1547.29 kJ/100 g. The range recorded in the study showed that the formulated blends are rich in carbohydrate content, implying that they can be used for the management of protein-energy malnutrition. The average energy required from complementary foods for developing countries (kJ/day) has been estimated at 840 (6–8 months), 1260 (9–11 months), and 1260 (12–23 months)^26,27^. Bello et al.^21^ reported that complex carbohydrates instead of sugars should provide more than half of the energy required for children as stipulated by the Food and Nutrition Board (1989). An adequate quantity of carbohydrates can be used to derive energy and spare protein, while the protein in the blends can be used for its primary function, which is building the body and repairing worn-out tissues instead of serving as a source of energy^28,29^.

**Table 1:** Proximate composition of the raw materials used for production sweet potato-cowpea-banana blends.

Proximate (%)	Banana	Cowpea	Orange fleshed sweet potato
Moisture	80.92 ± 0.002	9.31 ± 0.005	75.78 ± 0.002
Protein	1.83 ± 0.001	19.35 ± 0.031	2.33 ± 0.004
Fiber	1.46 ± 0.031	1.85 ± 0.005	1.02 ± 0.050
Fat	1.08 ± 0.015	0.92 ± 0.011	0.81 ± 0.010
Ash	1.93 ± 0.005	9.83 ± 0.006	1.49 ± 0.003
Carbohydrate	15.32 ± 0.001	58.74 ± 0.001	18.57 ± 0.010

n = 3 (analysis done in triplicates).

**Table 2:** Formulation ratio for the sweet potato-cowpea-banana (PCB) blends.

Sample code	OFSP (g)	Cowpea (g)	Banana (g)	Sugar (g)
PCB_1_	50	30	20	–
PCB_2_	55	30	15	–
PCB_3_	60	30	10	–
PCB_4_	65	30	5	–
PCB_5_	60	40	–	–
PCB_6_	50	30	15	5
PCB_7_	55	30	15	5
PCB_8_	60	30	5	5

OFSP = orange fleshed sweet potato.

**Table 3:** Nutrient composition of the sweet potato-cowpea-banana (PCB) blends.

Sample code	Moisture content (% w.b)	Ash (%)	Fiber (%)	Fat (%)	Protein (%)	Carbohydrate (%)	Calorific value (kJ/100 g)	Beta carotene (μg /100 g)	Vitamin A (μg /100 g)	Lycopene (mg/100 g)
PCB_1_	3.69 ± 0.076^c^	4.11 ± 0.113^ab^	3.38 ± 0.314^e^	0.82 ± 0.231^c^	10.19 ± 0.127^a^	77.89 ± 0.281^b^	1500.78 ± 13.203^c^	0.15 ± 0.001^a^	7.55 ± 0.096^a^	78.21 ± 0.026^e^
PCB_2_	3.58 ± 0.74^c^	4.61 ± 0.206^c^	2.24 ± 0.063^ab^	0.68 ± 0.422^bc^	10.04 ± 0.099^a^	78.85 ± 0.955^d^	1508.22 ± 0.435^cd^	0.15 ± 0.003^a^	7.62 ± 0.010^b^	78.39 ± 0.437^a^
PCB_3_	3.01 ± 0.029^d^	4.43 ± 0.188^c^	2.49 ± 0.804^bc^	0.67 ± 0.052^bc^	9.82 ± 0.087^ab^	79.58 ± 0.527^b^	1483.19 ± 10.97^b^	0.19 ± 0.005^c^	8.06 ± 0.024^e^	84.07 ± 0.032^f^
PCB_4_	3.04 ± 0.178^e^	5.14 ± 0.345^d^	2.30 ± 0.374^ab^	0.62 ± 0.031^a^	9.98 ± 0.030^ab^	78.92 ± 0.041^a^	1331.36 ± 3.119^a^	0.24 ± 0.001^d^	8.35 ± 0.025^g^	92.14 ± 0.859^d^
PCB_5_	2.75 ± 0.077^b^	4.64 ± 0.063^c^	1.96 ± 0.162^ab^	0.88 ± 0.087^a^	10.52 ± 0.130^a^	79.25 ± 0.280^b^	1520.31 ± 1.013^d^	0.17 ± 0.001^ab^	7.86 ± 0.005^d^	83.35 ± 0.234^b^
PCB_6_	3.39 ± 0.033^c^	4.58 ± 0.006^c^	1.74 ± 0.068^a^	0.62 ± 0.131^ab^	9.77 ± 0.422^bc^	79.90 ± 0.115^c^	1515.80 ± 3.334^d^	0.15 ± 0.007^ab^	7.62 ± 0.015^b^	81.25 ± 0.515^g^
PCB_7_	3.80 ± 0.261^a^	4.36 ± 0.133^c^	2.15 ± 0.111^ab^	0.75 ± 0.128^bc^	9.03 ± 0.058^bc^	80.33 ± 0.606^d^	1547.29 ± 5.497^e^	0.16 ± 0.009^bc^	7.77 ± 0.024^c^	83.07 ± 0.083^h^
PCB_8_	2.44 ± 0.110^b^	4.04 ± 0.083^a^	3.09 ± 0.161^cd^	0.64 ± 0.099^a^	8.46 ± 0.01^d^	81.33 ± 0.233^cd^	1515.62 ± 1.045^d^	0.21 ± 0.001^abc^	8.14 ± 0.011^f^	88.06 ± 0.050^c^

Codes: PCB_1_: 50% OFSP, 30% cowpea, 20% ripe banana flour and 0% sugar; PCB_2_: 55% OFSP, 30% cowpea, 15% ripe banana flour and 0% sugar; PCB_3_: 60% OFSP, 30% cowpea, 10% ripe banana flour and 0% sugar; PCB_4_: 65% OFSP, 30% cowpea, 5% ripe banana flour and 0% sugar, PCB_5_: 60% OFSP, 40% cowpea, 0% ripe banana flour and 0% sugar; PCB_6_: 50% OFSP, 30% cowpea, 15% ripe banana flour and 5% sugar; PCB_7_: 55% OFSP, 30% cowpea, 15% ripe banana flour and 5% sugar; PCB_8_: 60% OFSP, 30% cowpea, 5% ripe banana flour and 5% sugar.

^a–h^Values with different letters along a column for a given parameter are significantly different from each other at *P* ≤ 0.05.

PCB_7_ blend had the highest calorific value. The beta-carotene content ranged from 0.15 to 0.24 mg/100 g. PCB_4_ had the highest beta-carotenoid content, while PCB_1_, PCB_2_, and PCB_6_ (0.15) had the lowest value. The carotenoid content of the study is higher than that recorded by Kolawole et al.^30^ during the production of orange-fleshed sweet potato pasta. This may be due to alterations in the enzymatic oxidation and variations in the retention of carotenoids during processing. OFSP has also been reported as an excellent novel source of β-carotene which is a natural health-promoting compound^8^. The vitamin A content of the blends varied between 7.55 and 8.35 mg/100 g. The PCB_4_ blend showed the highest vitamin A value. This is due to an increase in the OFSP proportion. The lycopene content of the blends varied between 78.21 and 92.14 mg/100 g. PCB_4_ blend had the highest lycopene value.

### Color properties of the formulated sweet potato-cowpea-banana (PCB) blends

**Figure 1:**
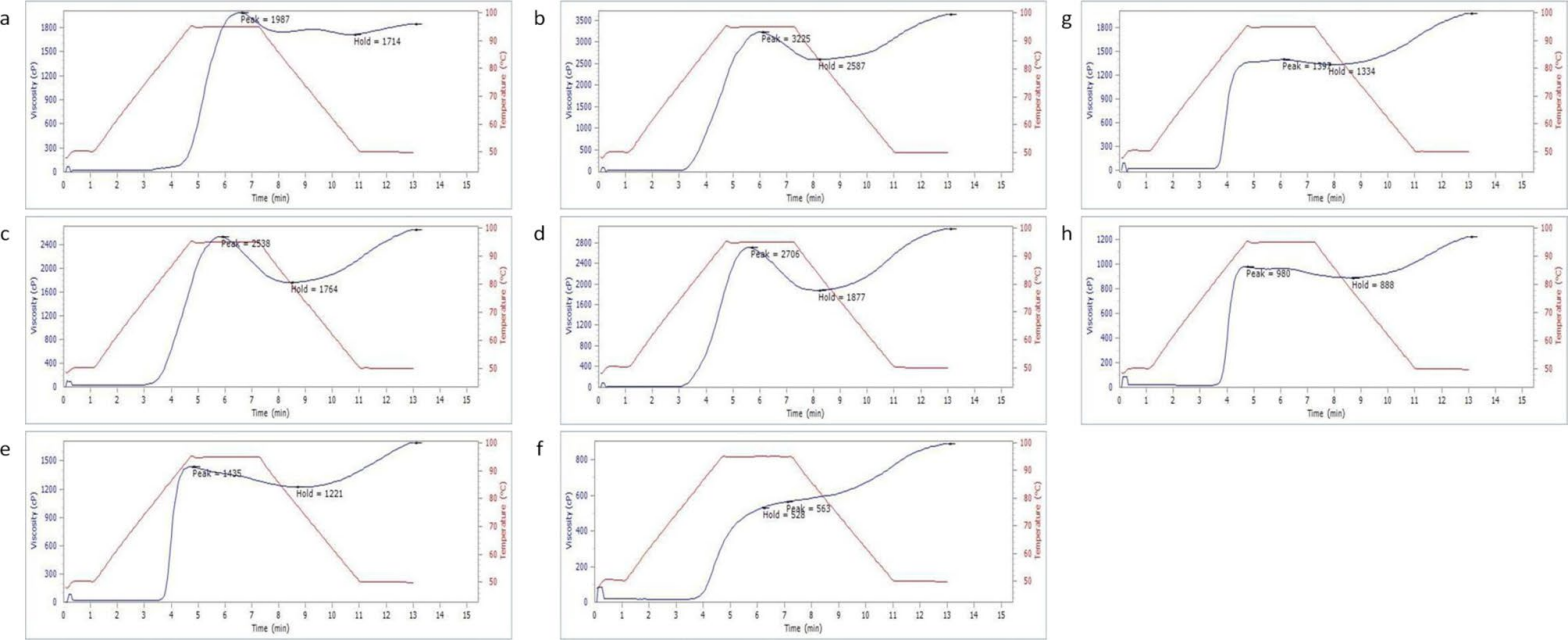
Pasting profile of the sweet potato-cowpea-banana (PCB) blends. (**a**) PCB1; (**b**) PCB2 (**c**) PCB3; (**d**) PCB4; (**e**) PCB5; (**f**) PCB6; (**g**) PCB7; (**h**) PCB8

The color evaluation of the PCB blends is shown in Fig. 1. There was some significance (*P* < 0.05) in the color parameters for the formulated blends. The lightness value (L) ranged from 42.14 to 67.18. PCB_4_ blend showed the highest L-value, while PCB_2_ presented the lowest L-value. This is due to an increase in the OFSP content of the PCB_4_ blend. The red-green value (a) was highest in the PCB_4_ blend and lowest in the PCB_6_ blend. This is also a result of the PCB_4_ blend having the highest OFSP content. The yellow-blue value (b) ranged from 19.37 to 29.44. PCB_4_ showed the highest b-value. The huge angle (HA) of the blends showed a small variation (1.33 to 1.47). The maximum HA was recorded for the PCB_6_ blend, while the lowest HA was shown in PCB_3_, followed by PCB_4_. This is expected since the HA reflects the ratio of the b to a value. The chroma value ranged from 13.44 to 24.14. PCB_4_ had the highest chroma value, while PCB_7_ showed the lowest, followed by PCB_2._

The color intensity (CI) varied between 36 and 53. 36 PCB_4_ blend presented the lowest CI, while PCB_2_ had the highest CI. Generally, the objective color evaluation of the blends showed that PCB_4_ presented significantly (*P* < 0.05) higher lightness and chroma and the lowest CI and HA. The high lightness values in the study can be attributed to the quantity of OFSP used for the formulation of the blends. OFSP flour has been reported to be high in carotenoid which is rich in β-carotene pigments^30^.

### Functional and pasting properties of the formulated sweet potato-cowpea-banana (PCB) blends

**Figure 2:**
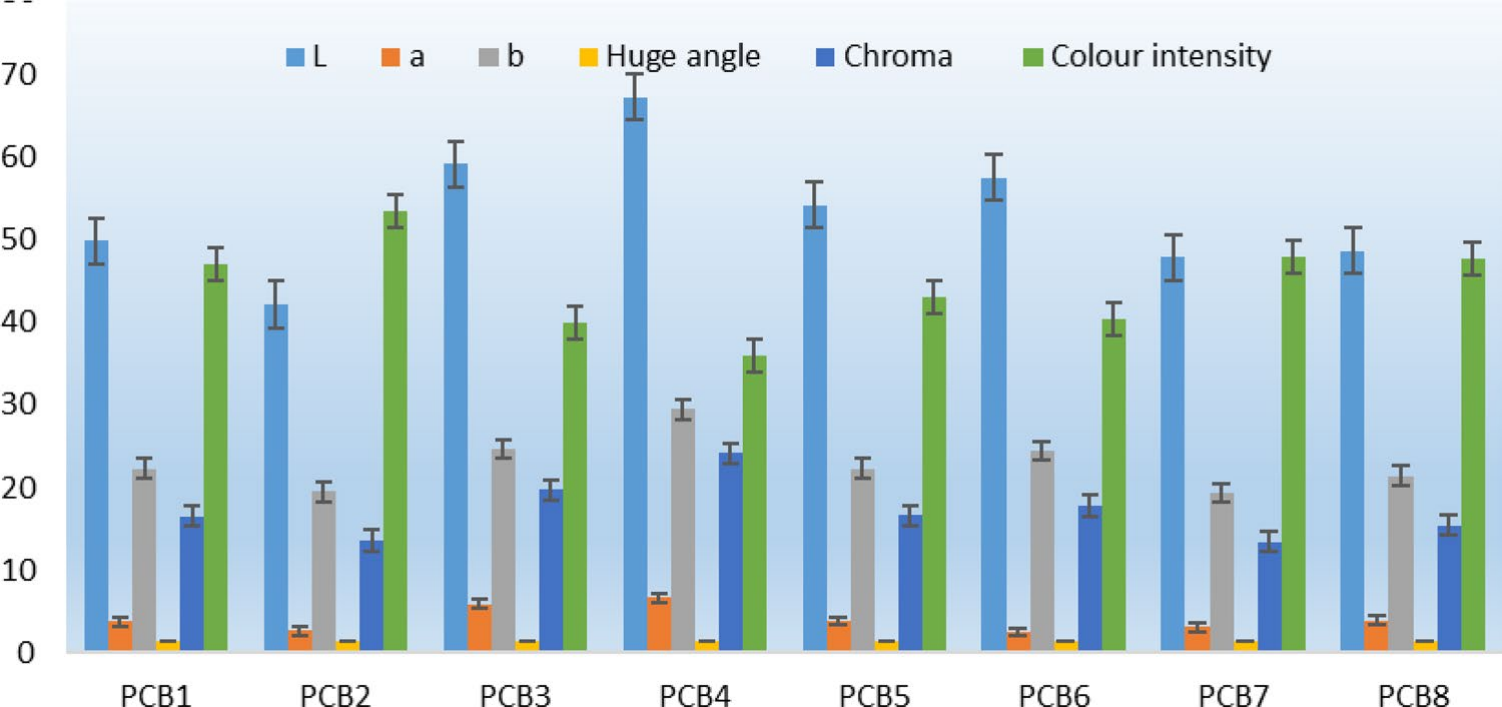
Color properties of the sweet potato-cowpea-banana (PCB) blends. Codes: PCB_1_: 50% OFSP, 30% cowpea, 20% ripe banana flour and 0% sugar; PCB_2_: 55% OFSP, 30% cowpea, 15% ripe banana flour and 0% sugar; PCB_3_: 60% OFSP, 30% cowpea, 10% ripe banana flour and 0% sugar; PCB_4_: 65% OFSP, 30% cowpea, 5% ripe banana flour and 0% sugar, PCB_5_: 60% OFSP, 40% cowpea, 0% ripe banana flour and 0% sugar; PCB_6_: 50% OFSP, 30% cowpea, 15% ripe banana flour and 5% sugar; PCB_7_: 55% OFSP, 30% cowpea, 15% ripe banana flour and 5% sugar; PCB_8_: 60% OFSP, 30% cowpea, 5% ripe banana flour and 5% sugar.

The result of the functional and pasting properties of the blends is represented in Table 4 and Fig. 2. There was a significant difference (*P* < 0.05) in the functional and pasting properties of the blends. The water absorption capacity (WAC) of the blends ranged from 189.73% to 226.54%. PCB_4_ blend showed the highest WAC, while PCB_6_ had the lowest WAC. WAC of flour is usually enhanced by some major chemical composition like protein and carbohydrate. The higher WAC of the PCB_4_ blend is due to its high starch composition and also depends on the availability of hydrophilic groups that hold water molecules and, on the gel, the forming ability of macromolecules^31^. Though the low water absorption capacity of the flour blend is desirable for producing a less bulky, thinner gruel with a high caloric density per unit value^31,32^.

**Table 4:** Functional and pasting properties of the sweet potato-cowpea-banana (PCB) blends.

Sample code	WAC (%)	PV (cP)	TV(cP)	BDV(cP)	FV(cP)	SBV(cP)	PT (min)	PAT (^o^C)
PCB_1_	200.51 ± 1.395^b^	1981.33 ± 13.429^e^	1724.00 ± 10.536^e^	273.00 ± 5.000^e^	1851.67 ± 7.638^d^	136.33 ± 6.506^a^	6.56 ± 0.031^e^	92.14 ± 0.859f.
PCB_2_	202.91 ± 2.517 ^bc^	3221.33 ± 10.017^h^	2588.00 ± 4.583^h^	637.67 ± 8.505 f.	3621.33 ± 10.263^h^	1040.67 ± 16.623^g^	6.10 ± 0.100^d^	78.39 ± 0.437^a^
PCB_3_	210.42 ± 2.542^cd^	2534.00 ± 8.718 f.	1762.67 ± 6.110 f.	771.33 ± 2.517^g^	2651.33 ± 7.638 f.	888.00 ± 6.557 f.	5.78 ± 0.076^c^	81.25 ± 0.515^b^
PCB_4_	226.54 ± 2.110^e^	2701.67 ± 4.041^g^	1873.33 ± 4.726^h^	829.67 ± 4.041^h^	3060.00 ± 26.458^g^	1190.00 ± 3.606^h^	5.60 ± 0.050^b^	78.21 ± 0.026^a^
PCB_5_	226.51 ± 6.032^e^	1432.33 ± 8.327^d^	1222.33 ± 5.132^c^	214.33 ± 4.509^d^	1693.67 ± 5.132^c^	472.00 ± 7.211^d^	4.73 ± 0.015^a^	83.35 ± 0.234^c^
PCB_6_	189.73 ± 3.898^a^	566.33 ± 10.408^a^	528.00 ± 4.000^a^	33.33 ± 2.082^a^	892.33 ± 5.132^a^	360.67 ± 2.517^c^	7.17 ± 0.153 f.	88.06 ± 0.050^e^
PCB_7_	207.23 ± 8.495 ^bcd^	1395.00 ± 5.292^c^	1332.33 ± 1.528^d^	63.00 ± 2.000^b^	1980.33 ± 2.082^b^	648.67 ± 6.028^e^	6.09 ± 0.179^d^	83.07 ± 0.083^c^
PCB_8_	213.74 ± 1.924^d^	979.33 ± 3.055^b^	887.33 ± 2.082^b^	90.67 ± 1.528^c^	1221.00 ± 3.606^c^	330.33 ± 2.082^b^	4.67 ± 0.025^a^	84.07 ± 0.032^d^

Codes: WAC = water absorption capacity, PV = peak viscosity, TV = trough viscosity, FV = final viscosity, SBV = setback viscosity, PT = peak time, PAT = pasting temperature; PCB_1_: 50% OFSP, 30% cowpea, 20% ripe banana flour and 0% sugar; PCB_2_: 55% OFSP, 30% cowpea, 15% ripe banana flour and 0% sugar; PCB_3_: 60% OFSP, 30% cowpea, 10% ripe banana flour and 0% sugar; PCB_4_: 65% OFSP, 30% cowpea, 5% ripe banana flour and 0% sugar, PCB_5_: 60% OFSP, 40% cowpea, 0% ripe banana flour and 0% sugar; PCB_6_: 50% OFSP, 30% cowpea, 15% ripe banana flour and 5% sugar; PCB_7_: 55% OFSP, 30% cowpea, 15% ripe banana flour and 5% sugar; PCB_8_: 60% OFSP, 30% cowpea, 5% ripe banana flour and 5% sugar.

^a–h^Values with different letters along a column for a given parameter are significantly different from each other at *P* ≤ 0.05.

The peak viscosity (PV) of the blends ranged from 566.33 to 3221.33 cP. PCB_2_ blend showed the highest PV, followed by PCB_4_, while PCB_6_ presented the lowest PV. This shows that PCB_2_ and PCB_4_ had a higher water-binding capacity, quality, and ease of granular disintegration. This increase is due to the higher content of OFSP and bananas due to their higher carbohydrate content and the absence of an additive (sugar). High peak viscosity is responsible for high swelling capacity due to the availability of more starch granules^31^.

The trough viscosity (TV) varied between 528 and 2588 cP. PCB_2_ and PCB_4_ had the highest TV, while PCB_6_ showed the lowest TV, followed by PCB_8_. High TV shows the stability characteristics of the blend during heating and cooling. This is as a result of the increased inclusion of the OFSP (i.e., a carbohydrate source). The breakdown viscosity (BDV) of the blends ranged from 33.33 to 829.67 cP. The least BDV was for the PCB_6_ blend, while the highest was for the PCB_4_ blend, followed by PCB_3_ (771.33 cP) and PCB_2_ (637.67 cP). The breakdown viscosity is the measure of the degree of paste strength or starch granule fragmentation during heating^33^. Therefore, the weaning mix with the high breakdown viscosity will have a more stable paste during heating than others with a low breakdown viscosity^34^. The final viscosity (FV) of the blends varied between 892.33 and 3621.33 cP. PCB_2_ blend had the highest FV; it was closely followed by PCB_4_ blend (3060 cP), while PCB_6_ blend presented the lowest FV. High FV represents a higher ability of the blend to paste during heating, low viscosity connotes thinner gruel which is a desirable quality because the viscosity of weaning or complementary food plays a significant role in its acceptability as well as an infant’s energy intake^34^. The final viscosity is often regarded as an indicator of the stability of the cooked paste when prepared^31^; the setback viscosities (SBV) of the blends ranged from 136.33 to 1190 cP. PCB_4_ showed the highest SBV, while PCB_1_ presented the lowest SBV. A higher SBV shows the ability of the cooked blend to retrograde during cooling. PCB_6_ and PCB_8_ had the lowest SBV values (360.67 and 333.33), which indicate low starch retrogradation and syneresis^33^. Peak time (PT) varied between 4.67 and 7.17 min. Higher PT was shown for the PCB_6_ blend, while lower PT was recorded for the PCB_8_ (4.67 min), PCB_5_ (4.73 min), and PCB_4_ (5.6 min). The higher PT reflects that more time is required for complete gelatinization of the starchy blends. The pasting temperature (PAT) ranged from 78.21 to 92.14 °C and the variances in the pasting temperatures of the blends of weaning food show that they all have different gelatinization temperatures. It also suggests the minimum temperature required to cook a given sample, which could also have effects on energy usage^35^. PCB_4_ blend showed a lower PAT, while PCB_1_ blend had a higher PAT, though a lower PAT is still desirable. Generally, the functional and pasting evaluation showed that PCB_4_ (65 g of OFSP, 30 g of cowpea, and 5 g of ripe banana flour) showed significantly higher water absorption capacity, peak viscosity, trough viscosity, breakdown viscosity, final viscosity, setback viscosity, and lower peak time and pasting temperature. Functional properties of the formulated blends in the study showed reduction in water absorption capacity, and pasting properties which is advantageous as this would assist in the preparation of gruels with low viscosity and high calorie density per unit volume that can be easily swallowed by babies^21^.

### Relationship between color and nutritional properties of the formulated sweet potato-cowpea-banana (PCB) blends

The correlation between the color parameters and some nutritional compositions is represented in Table 5. Beta-carotene showed a strong positive significant (*P* < 0.05) correlation with vitamin A (0.84**), lycopene (0.56**), L-value (0.65**), a-value (0.68**), b-value (0.58**), and chroma (0.62**), but a negative correlation with HA (− 0.59**) and CI (− 0.61**). This signifies that increase in the proportion of Beta-carotene raw material source will consequently increase the vitamin A, lycopene, L-value, a-value, b-value, chroma and decrease the HA and CI. Vitamin A showed similar positive and negative correlations as beta-carotene. Lycopene had no significant (P > 0.05) correlation with any of the color parameters, indicating that its increase or decrease will not affect the color of the blends. The L-value presented a strong significant correlation with HA (− 0.58**), chroma (0.93**) and CI (− 0.99**). Similar trend was noticed for a and b-value. This study's correlation between colour parameters and some nutritional components, notably lycopene, beta-carotene, and vitamin A, supported earlier researches suggesting that colour has a significant role in drawing children's attention to food while preserving the flour mix's optimal nutritional value^8,9,18,20^.

**Table 5:** Relationship between nutritional and colour properties of the potato-cowpea-banana (PCB) blends.

	Beta carotene	Vitamin A	Lycopene	L	a	b	Huge angle	Chroma	Colour intensity
Beta carotene	1								
Vitamin A	0.84**	1							
Lycopene	0.56**	0.72**	1						
L	0.65**	0.50*	0.32	1					
A	0.68**	0.64**	0.31	0.78**	1				
B	0.58**	0.39	0.17	0.93**	0.76**	1			
Huge angle	− 0.59**	− 0.60**	− 0.31	− 0.58**	− 0.94**	− 0.51*	1		
Chroma	0.62**	0.46*	0.21	0.93**	0.85**	0.99**	− 0.63**	1	
Colour intensity	− 0.61**	− 0.45*	− 0.34	− 0.99**	− 0.71**	− 0.87**	0.53**	− 0.87**	1

*moderate positive relationship, **fairly strong positive relationship, ***very strong positive relationship

### Relationship between proximate and pasting properties of the formulated sweet potato-cowpea-banana (PCB) blends

The Pearson correlation between proximate and pasting properties is shown in Table 6. The ash content of the blends significantly correlated (*P* < 0.05) with fiber (− 0.61**), fat (− 0.41*), carbohydrate (− 0.61**), calorific value (− 0.67**), BDV (0.52**), FV (0.45*), SBV (0.67**), and PAT (− 0.52**). This signifies that increasing the raw materials with high ash content will decrease the fiber, fat, carbohydrate, calorific value, and PAT and increase the BDV, FV, and SBV. Fiber content only correlated well with protein (0.61**). Fat content of the blends showed a positive correlation with protein (0.49*) and PT (0.47*), showing that an increase in the raw material with a high fat content will increase the time required for complete gelatinization of the blend. Protein content showed a strong and significant correlation with SBV (− 0.52**) and PAT (0.72**), showing that an increase in protein source will decrease the tendency of the cooked blend to retrograde during cooling and increase the gelatinization temperature. Carbohydrate correlated well with energy content (0.98**), WAC (− 0.44*), BDV (− 0.62**), and SBV (− 0.53**), indicating that an increase in the carbohydrate source will increase the energy content dissipated by the blend, decrease its WAC, and increase the ability of the starch to disintegrate during heating and syneresis. Amongst the pasting parameters there were significant correlation coefficients. In order to improve the health, mineral content, and nutritional values of the developed flour mix as complementary food, it is important to consider the relationship between proximate and pasting properties. Specifically, an increase in protein source will decrease the cooked blend's tendency to retrograde this is agreement with findings of previous researches^2,4,9^.

**Table 6:** Correlation between proximate and pasting properties of the sweet potato-cowpea-banana (PCB) blends.

	Moisture	Ash	Fiber	Fat	Protein	CHO	Calorific	WAC	PV	TV	BDV	FV	SBV	PT	PAT
Value															
Moisture	1														
Ash	0.70**	1													
Fiber	− 0.04	− 0.61**	1												
Fat	− 0.29	− 0.41*	0.17	1											
Protein	− 0.11	− 0.42*	0.61**	0.49*	1										
CHO	− 0.98**	− 0.61**	− 0.06	0.27	0.01	1									
Calorific Value	− 0.99**	− 0.67**	− 0.04	0.36	0.09	0.98**	1								
WAC	0.46*	0.38	0.04	− 0.41	− 0.30	− 0.44*	− 0.49*	1							
PV	0.49*	0.37	0.11	0.25	0.08	− 0.40	− 0.48*	0.24	1						
TV	0.34	0.26	0.15	0.35	0.18	− 0.25	− 0.33	0.16	0.97**	1					
BDV	0.72**	0.52**	0.02	0.02	− 0.10	− 0.62**	− 0.71**	0.36	0.90**	0.77**	1				
FV	0.49*	0.45*	− 0.03	0.21	− 0.09	− 0.38	− 0.47*	0.26	0.97**	0.95**	0.86**	1			
SBV	0.64**	0.67**	− 0.33	− 0.07	− 0.52**	− 0.53**	− 0.61**	0.37	0.76**	0.66**	0.82**	0.86**	1		
PT	− 0.03	0.03	− 0.15	0.47*	0.37	− 0.001	0.09	− 0.79**	− 0.02	0.01	− 0.08	− 0.04	− 0.1	1	
PAT	− 0.45*	− 0.52**	0.34	0.23	0.72**	0.32	0.43*	− 0.52**	− 0.60**	− 0.51*	− 0.65**	− 0.72**	− 0.91**	0.41*	1

WAC = water absorption capacity, PV = peak viscosity, TV = trough viscosity, FV = final viscosity, SBV = setback viscosity, PT = peak time, PAT = pasting temperature, CHO = carbohydrate.

### Acceptability of the formulated sweet potato-cowpea-banana (PCB) blends

**Figure 3:**
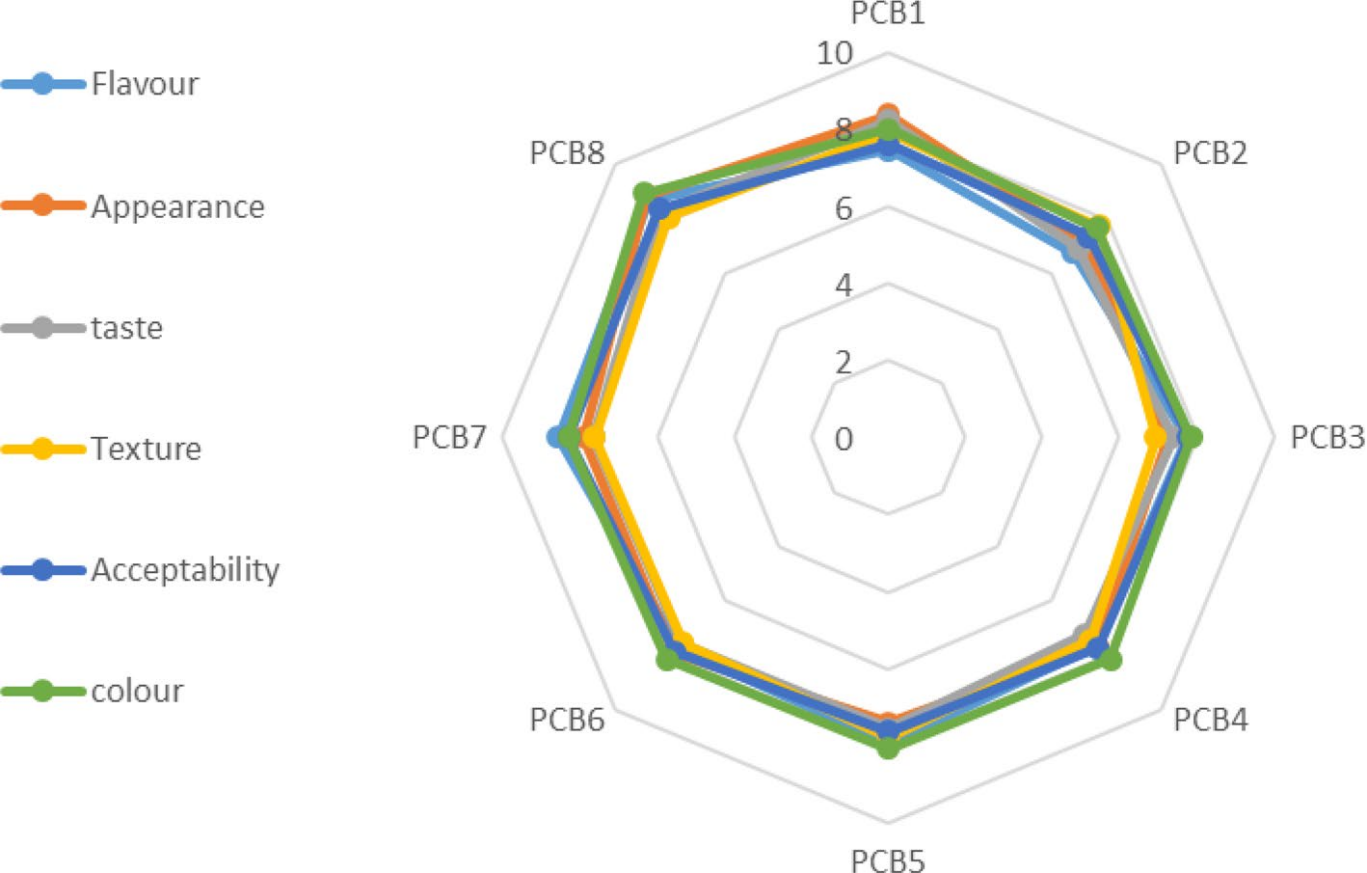
Sensory evaluation of porridge containing sweet potato-cowpea-banana blends (PCB). Codes: PCB_1_: 50% OFSP, 30% cowpea, 20% ripe banana flour and 0% sugar; PCB_2_: 55% OFSP, 30% cowpea, 15% ripe banana flour and 0% sugar; PCB_3_: 60% OFSP, 30% cowpea, 10% ripe banana flour and 0% sugar; PCB_4_: 65% OFSP, 30% cowpea, 5% ripe banana flour and 0% sugar, PCB_5_: 60% OFSP, 40% cowpea, 0% ripe banana flour and 0% sugar; PCB_6_: 50% OFSP, 30% cowpea, 15% ripe banana flour and 5% sugar; PCB_7_: 55% OFSP, 30% cowpea, 15% ripe banana flour and 5% sugar; PCB_8_: 60% OFSP, 30% cowpea, 5% ripe banana flour and 5% sugar.

Sensory responses to the taste, smell, and texture of foods assist in determining food preferences and eating habits. Color is a strong indicator of whether the developed product will be acceptable. The color of the sweet potato-cowpea banana blends presented to panelists in porridge form ranged from 8.96 to 7.68 as assessed based on a 9-point hedonic scale (Fig. 3), and they were generally acceptable, with the highest average score of 8.96 recorded for PCB_8_ blends (60:30:5:5 of OFSP, cowpea, ripe banana, and sugar, respectively) and the least (7.68) for PCB1 blends (50:30:20:0 of OFSP, cowpea, ripe banana, and sugar, respectively). Flavor is also a significant element in the acceptance of foods as it’s a combination of the senses of taste, aroma, and mouth feel. There was a significant difference in the flavor of the blends, with PCB_8_ (8.75) being most preferred, followed by PCB_7_ (8.57), PCB_5_ (7.93), PCB_3_ (7.75), PCB_6_ (7.71), PCB_4_ (7.64), PCB_1_ (7.46), and PCB_2_ (6.79) in descending order. The panelists rated the appearance (8.88–7.21) in a similar order, except that PCB_6_ and PCB_7_ have the same score (7.89); besides that, there is no significant difference between PCB_5_ (7.39), PCB_3_ (7.24), and PCB_2_ (7.21), respectively. There were no significant differences texture and the tastes of all the sweet potato-cowpea-banana (PCB) blends assessed as were all acceptable range with PCB_8_ most preferred. Overall acceptability of the blend was highly acceptable with the lowest been 6.36 and highest been 7.95. There is no negative report with all sample. Addition of banana and cowpea to OFSP up to 40 and 20% reveal no undesirable descriptions in the developed products. The general observation that PCB8 is more preferred compared to PCB_1_ agrees with Biernacka et al.^20^, who reported that overripe banana flour's natural sweetness with little sugar increases the overall acceptability of muffins. Products such as pasta, biscuits, and porridge from OFSP have been reported to be acceptable in terms of color, texture, taste, flavor, and texture^8,30^.

In conclusion, the nutritional compositions of the formulated blends show that they can be used in the management of protein-energy malnutrition. The formulated blends showed advantages over other blends recorded in the literature as they had good water absorption capacity. PCB_4_ in particular was noted to be the best in terms of its vitamin A value, functional properties, and pasting properties, as it had higher WAC, PV, TV, BV, FV, and SV. This study is showcasing locally obtained food items for innovative combination of tubers-pulses and banana for production of a nutritionally enriched flour mix with potential of combating vitamin A deficiency (VAD) in the country.

## Materials and methods

### Materials

Freshly harvested OFSP (*Ipomoea batatas*) and bunches of ripe sweet bananas (*Musa acuminate*) were transported to the food processing laboratory in sacks and Ife brown variety cowpea (*Vigna unguiculata*) were procured from a local vendor in Omu-Aran, Nigeria. Table 1 shows the proximate analysis of the raw materials used for production of sweet potato-cowpea-banana blends.

### Production of sweet potato-cowpea-banana blends

Sorted cowpea seeds were weighed (5 kg) and soaked in 10 L of potable water for 6 h. The soaked seeds were drained, manually dehulled, and washed under running water. Four (4) kg of cleaned dehulled cowpea was steamed in 2 L of boiling water for 15 min, allowed to cool and dried in a hot air oven for 12 h at 60 °C, and kept for further processing. Washed and peeled OFSP tubers were diced (3 mm) and dried on stainless steel trays at 60 °C for 12 h^2^. The modified method of (36) was used for the preparation of dried banana chips. Ripe bananas were peeled, sliced into thin sheets (2 mm) using a slicer; 0.5% (v/w) of fresh lime juice were added to reduce browning of the chips during drying and dried at 60^0^C for 24 h. Eight (8) different formulations of the sweet potato-cowpea-banana (PCB) blends were carried out as shown in Table 2 and properly labeled samples were packaged and sealed in 100 g sachets in triplicates for further analysis. All methods were carried out in accordance with relevant guidelines and regulations. In addition, all experimental protocols were approved by Landmark University ethical committee (LUAC/FSN/SCI/0001).

### Acceptability assessment of sweet potato-cowpea-banana blends

Eight sweet potato-cowpea-banana blends flour samples were prepared into porrigde. The porrigde samples were prepared Blended sweet potato, cowpea, and banana Reconstituted porridge was made by mixing 50 g of flour with 250 mL of portable water, then adding boiling water and stirring constantly until gelatinization took place. and 50 ml randomly served in a labelled cups to 60 members of a panel conversant (nursing mothers) with commercial complementary food in coded plates, for evaluation using a 9 point structured Hedonic scale with 9 synonymous to like extremely and 1 dislike extremely. The colour, appearance, aroma, smoothness, and overall acceptability were assessed and scored. Inclusion criteria requires the recruited panelist must be a nursing mother or mothers with toddlers within the ages 6 months to 5 year. The participation is voluntary coupled with willingness to participate, availability and freedom from food allergies. Panelist must be familiar with the raw materials used for production and the final product. Those who participated in sensory tests are willing to use product as potential consumers based on their acceptability. While those who did not meet the inclusion criteria were excluded as well as those subjects who have prior technical knowledge of products or projects were not allowed to participate in sensory testing^31^.

### Colour analysis of sweet potato-cowpea-banana (PCB) blends

Using a bench-top spectrophotometer Colorflex-EZ (A60-1014–593, Hunter Associates, Reston, VA, USA); colour of sweet potato-cowpea-banana (PCB) blends were measured in terms of lightness (L*) and color values (+ a: red; -a: green; + b: yellow; -b: blue) as described by^36^.

$${\text{Hue angle }}\left( {{\text{H}}*} \right) \, = {\text{ arctan }}\left( {{\text{b}}*/{\text{a}}*} \right)$$

$${\text{Chroma}}, \, \left( {{\text{C}}*} \right) \, = \sqrt {{\text{a}}^{2} + {\text{b}}^{2} }$$

$${\text{Hue}}\;{\text{angle}} = \tan^{ - 1} \left( {{\text{b}}/{\text{a}}} \right)$$

$${\text{Total}}\,{\text{color}}\,{\text{difference}}\left( {\Delta {\text{E}}*} \right) \, = \sqrt {\left( {\Delta {\text{L}}*} \right)^{2} + \left( {\Delta {\text{a}}*} \right)^{2} \left( {\Delta {\text{b}}*} \right)^{2} }$$

Differences of L*, a* and b* were used to calculate the changes in different color attributes of samples.

$$\Delta {\text{L}}* \, = L* \, - \, L$$

$$\Delta a* = a* - a$$

$$\Delta b* = b* - b$$

where *L, a, b* is color component values of control. The following values were used to determine if the total color difference was visually obvious^36^.

∆E* < 1 = color differences are not obvious for the human eye.

1 < ∆E* < 3 = color differences are not appreciative by the human eye.

### Sweet potato-cowpea-banana (PCB) blends proximate composition determination

Sweet potato-cowpea-banana (PCB) blends proximate composition was determined using the procedure of AOAC, (2019) for crude fat, protein, fiber, ash, and moisture contents. Total carbohydrate was calculated by difference and calorific value were calculated and documented in kJ/100 g.

### β-Carotene and lycopene estimation in sweet potato-cowpea-banana blends

Beta-carotene and Lycopene of sweet potato-cowpea-banana (PCB) blends were determined using dried methanolic extract Olaniran et al.^37^. 100 mg of extract was mixed with 10 ml of an acetone-hexane mixture (4:6) for 1 min and filtered. The absorbance was recorded at three different wavelengths 453, 505, and 663 nm respectively, and calculated using the formula:

$${\text{Beta}} - {\text{Carotene }}\left( {{\text{mg}}/{1}00{\text{ml}}} \right) \, = \, 0.{\text{216 X A663 }}{-} \, 0.{3}0{\text{4 X A5}}0{5 } + \, 0.{\text{452 X A453}}$$

$${\text{Lycopene }}\left( {{\text{mg}}/{1}00{\text{ml}}} \right) \, = - 0.0{\text{458 X nA663 }} + \, 0.{\text{372X A5}}0{5 } - \, 0.0{8}0{\text{6 X A453}}$$

where A = absorbance.

### Sweet potato-cowpea-banana (PCB) blends Vitamin A quantification

One (1) gram of sweet potato-cowpea-banana (PCB) blends samples were homogenized and saponified with 5 ml of 12% alcoholic potassium hydroxide in a water bath for 30 min at 60 °C. The saponified extract was transferred into a separating funnel and thoroughly mixed with 15 ml of petroleum ether. The lower aqueous layer was transferred to a new separating funnel followed by a collection of the upper petroleum ether layer containing the carotenoids. Extraction was repeatedly done till the aqueous layer became colorless. Anhydrous sodium sulphate was added to the petroleum ether extract to remove excess moisture. The absorbance of the yellow color was read in a spectrophotometer at 460 nm using petroleum ether as blank. The quantity of Vitamin A (beta-carotene equivalent) was calculated as 1 IU Vitamin A = 0.6 μg β-carotene, 1 IU Vitamin A = 0.3 μg vitamin A^38^.

### Pasting properties of sweet potato-cowpea-banana blends

Pasting properties were determined using Rapid Visco Analyzer (RVA). Three (3) grams of sweet potato-cowpea-banana (PCB) blends were weighed into a test canister and distilled water (5 ml) added. The paddle placed in the canister and the slurry was vigorously jogged using a blade as the analysis proceeds and terminated automatically. The heated slurry from 50 to 95 °C was allowed to cool to 50 °C by the thorough continuous stirring of the content using a plastic paddle within 12 min rotating the can at 160 rpm. Peak viscosity, setback viscosity, final viscosity, pasting temperature, pasting time, trough, and breakdown value were estimated^39^.

### Water absorption capacity of sweet potato-cowpea-banana-blends

Water absorption capacity (WAC) of the sweet potato-cowpea-banana (PCB) blends sample was determined by weighing 0.5 g of the sample dissolved in 10 ml of distilled water in centrifuge tubes and vortexed for 30 s. The dispersions were allowed to stand at room temperature for 30 min, centrifuged at 3000 rpm for 25 min. Fitration of resultant supernatant through filter paper (Whatman No 1) was carried out and volume recovered was correctly measured. Calculation of differences between initial volumes of distilled water added to the sample and the volume obtained after filtration was recorded. The results were reported as mL of water absorbed per gram of sample (ml/g)^31^.

### Statistical analysis

Analyses of the samples were conducted in triplicates. Analysis of variance (ANOVA) and Duncan's multiple range tests (*P* < 0.05) were conducted using IBM SPSS Statistics 22.

### Informed consent

Informed consent was obtained from all participants for this study.

### Supplementary Information

Supplementary Information.

## Data Availability

The datasets used and/or analysed during the current study available from the corresponding author on reasonable request.
