# Supplementary material for: Production, acceptability, nutritional and pasting properties of orange-flesh sweet potato, cowpea and banana flour mix
Source: Sci Rep. 2024 Feb 26;14:4602. doi: 10.1038/s41598-024-55312-1 (PMC10897140; doi:10.1038/s41598-024-55312-1)
Supplement: Supplementary file 1 — Supplementary Information. [file 41598_2024_55312_MOESM1_ESM.docx]

**Production, Acceptability, Nutritional and Pasting properties of Orange-Flesh Sweet Potato, Cowpea and Banana flour mix**

Abiola Folakemi Olaniran^1^*, Clinton Emeka Okonkwo^2^, Omorefosa Osarenkhoe Osemwegie^1^, Yetunde Mary Iranloye^1^, Adejoke Deborah Adewumi^2^, Abiola Ezekiel Taiwo^3^, Oluwakemi Christianah Erinle^2^, Iyanuoluwa Esther Ajayi^1^, Oluwafemi Adeleke Ojo^4^*

^1^Department of Food Science and Microbiology, P.M.B. 1001, College of Pure and Applied Sciences, Landmark University, Omu-Aran, Kwara State, Nigeria

^2^Department of Agricultural and Biosystems Engineering, College of Engineering, P.M.B. 1001, Landmark University, Omu-Aran, Kwara State, Nigeria

^3^Department of Chemical Engineering, Landmark University, Omu-Aran Kwara State, Nigeria

^4^Department of Biochemistry, Bowen University, Iwo, 232101, Osun State, Nigeria

*Corresponding author: AFO: [olaniran.abiola@lmu.edu.ng](mailto:olaniran.abiola@lmu.edu.ng); OAO: oluwafemiadeleke08@gmail.com

**Supplementary 1:** **Research Experimental design formulation of sweet potato-cowpea Banana blends**

Factor 1 Factor 2 Factor 3 Factor 4
 Std Run Block A:Potato B:Cowpea C:ripe banana D:Sugar Response 1
 g g g g
 25 1 Block 1 50.00 30.00 20.00 0.00
 6 2 Block 1 45.00 25.00 5.00 5.00
 4 3 Block 1 55.00 30.00 50.00 0.00
 10 4 Block 1 40.00 25.00 5.00 10.00
 15 5 Block 1 55.00 30.00 15.00 5.00
 8 6 Block 1 50.00 30.00 5.00 5.00
 7 7 Block 1 60.00 30.00 10.00 0.00
 21 8 Block 1 65.00 30.00 5.00 0.00
 13 9 Block 1 60.00 30.00 5.00 5.00
 3 10 Block 1 50.00 25.00 5.00 10.00
 5 11 Block 1 40.00 25.00 10.00 5.00
 22 12 Block 1 60.00 40.00 0.00 0.00
 9 13 Block 1 40.00 30.00 10.00 10.00
 19 14 Block 1 45.00 27.50 5.00 7.50
 23 15 Block 1 40.00 25.00 5.00 10.00
 14 16 Block 1 50.00 30.00 15.00 5.00
 11 17 Block 1 45.00 30.00 5.00 10.00
 17 18 Block 1 45.00 25.00 7.50 7.50
 24 19 Block 1 55.00 30.00 15.00 5.00
 1 20 Block 1 40.00 30.00 5.00 7.50
 12 21 Block 1 40.00 30.00 7.50 5.00
 2 22 Block 1 40.00 27.50 5.00 5.00
 18 23 Block 1 45.00 30.00 7.50 7.50
 20 24 Block 1 45.00 27.50 10.00 7.50
 16 25 Block 1 42.50 27.50 7.50 7.50 Factor 1 Factor 2 Factor 3
